# Individual differences in affect in response to physical activity

**DOI:** 10.3389/fpsyg.2025.1575189

**Published:** 2025-06-10

**Authors:** Shinji Takahashi, Yosuke Sakairi, Philip M. Grove

**Affiliations:** ^1^Department of Psychology and Behavioral Sciences, Faculty of Human Sciences, Tohoku Gakuin University, Sendai, Japan; ^2^Department of Psychology and Education, Faculty of Education, Tokoha University, Shizuoka, Japan; ^3^School of Psychology, The University of Queensland, Brisbane, QLD, Australia

**Keywords:** core affect, vigorous physical activity, mixed model, variance component, intraclass correlation coefficient

## Abstract

**Introduction:**

A single bout of physical activity can benefit one’s psychological state, increasing positive affect. Individual differences in these feelings are known to correlate with mental health; however, individual differences in response to physical activity are unclear. This study aimed to quantitatively evaluate individual differences in affect in response to acute physical activities. Quantifying those individual differences implicitly assumed in previous studies would facilitate understanding the relationship between physical activity adherence and mental health.

**Methods:**

The dataset comprised valence (pleasant-unpleasant) and arousal (active-inactive) measurements taken before and after two types of physical activities (running and badminton) with a crossover design. Valence and arousal were analyzed using a mixed model. Then, the intraclass correlation coefficients (ICCs) for valence and arousal, which are the ratio of the variance components of individual differences and the sum of total variance components, were calculated. Information processing in cognitive functions was also analyzed and compared variance components among valence, arousal, and information processing to comprehensively evaluate individual differences in valence and arousal in response to physical activity.

**Results and discussion:**

The results showed that individual differences in valence and arousal in response to physical activity were significant variance components, whereas the variance component in information processing was not significant. The ICCs for valence, arousal, and information processing were 0.603 (95% confidence interval [95%CI]: 0.430–0.769), 0.349 (95%CI: 0.202–0.512), and 0.171 (95% CI: 0.164–0.217), respectively, demonstrating that the ICC for valence is significantly more pronounced than that for information processing. These findings indicate that the effects of physical activity on affect vary among individuals, particularly regarding changes in valence. Considering individual differences is essential when tailoring physical activity treatments for health.

## 1 Introduction

There is substantial evidence that physical activity promotes not only physical but also mental health (Biddle et al., 2019; Carter et al., 2021; Nuzum et al., 2020). Recently, a scoping review by Pascoe et al. (2020) reported that increasing physical activity attenuates the symptoms of depression and anxiety in young people, suggesting that physical activity intervention is a promising strategy for mental health. A systematic review by Maynou et al. (2021) also found an inverse association between physical activity and mental health problems, reporting that the effects of physical activity on mental health are small to moderate. Numerous studies indicate that regular physical activity or physical activity intervention effectively promotes mental health (Biddle et al., 2019; Carter et al., 2021; Nuzum et al., 2020; Pascoe et al., 2020; Maynou et al., 2021).

Even a single bout of physical activity can benefit affect. A single bout of physical activity can rapidly increase neurotrophic factors such as brain-derived neurotrophic factor and insulin-like growth factor (Behrendt et al., 2021; Hung et al., 2018). These factors act on the prefrontal cortex, contributing to the modulation of hedonic tone (Deyama et al., 2022). In addition, physical activity elevates levels of peripheral and central norepinephrine (Basso and Suzuki, 2017), which enhances physiological and psychological arousal. These mechanisms may help explain the positive effects of acute physical activity on affect. Reed and Ones (2006) conducted a meta-analysis to examine the effect of a single bout aerobic physical activity on core affect, which is the elementary affective feeling defined as “A neurophysiological state that is consciously accessible as a simple, nonreflective feeling that is an integral blend of hedonic (pleasure–displeasure) and arousal (sleepy–activated) values” (Russell, 2003). They found that a single bout of aerobic physical activity moderates the increase of the positive-activated (pleasant and activated) affect. The acute effect of physical activity on core affect was also found in a longitudinal observation study. Bourke et al. (2022) reported that vigorous physical activity measured by an accelerometer was positively correlated with energetic arousal (pleasant and activated affect) for young people. Additionally, Bourke et al. revealed that vigorous physical activity indirectly increased life satisfaction, a component of subjective well-being (Diener, 1984), by mediating physical activity-induced energetic arousal. These findings indicate that even a single bout of physical activity positively increases core affect, contributing to good mental health and well-being.

Increasing core affect by acute physical activity is associated with mental health (Reed and Ones, 2006). Additionally, individual differences in core affect are correlated to personality traits and mental health (Kuppens et al., 2007; Timmermans et al., 2009). Kuppens et al. (2007) found that people who are neurotic and pessimistic exhibit more pronounced within-person variability of core affect compared to those with extraversion and optimism. Furthermore, the authors decomposed the within-person variabilities, elucidating that qualitative changes in the core affect, such as a shift from “enthusiastic” and “happy” (positive high arousal) to “sluggish” and “sad” (negative low arousal), contribute to depression symptoms and low self-esteem. Similarly, Timmermans et al. (2009) investigated the significance of the within-person variability of core affect for daily living. They found significant within-person variabilities of valence and arousal in response to daily events, suggesting that the within-person variabilities of core affect were associated with personality traits. People with high extraversion tended to experience higher arousal during no- and low-impact daily events (e.g., chatting with friends) compared to those with low extraversion. In contrast, during high-impact daily events (e.g., unexpected incidents), individuals with high extraversion were less aroused than those with low extraversion. Regarding valence, highly neurotic individuals tended to feel less pleasant during common daily events compared to those with low neuroticism. Previous research underscores the importance of individual differences in core affect for maintaining mental health and psychological well-being.

Considering the individual differences in core affect over time, it is plausible that there are also individual differences in core affect in responses to physical activity. That is, even if individuals engage in the same physical activities, the effects of physical activity may vary for each person. The meta-analysis by Reed and Ones (2006) found an effect size of acute aerobic physical activity for core affect of 0.47 accompanied by a large standard deviation (SD) of 0.37. They speculated that individual differences likely contribute to this large SD. The variability in effect size may stem from factors such as baseline core affect levels, exercise variables (e.g., intensity, duration, mode), and individual performance and fitness levels. These potential causes are primarily consistent with the thoughts of Herold et al. (2021), who discussed the individual differences in the association between neurocognition and acute physical activity. Because changes in cognitive function after a single bout of physical activity have been found to correlate with changes in core affect (arousal) (Byun et al., 2014), it is reasonable to assume that individual differences in affective responses may also be substantial. Supporting this, Takahashi and Grove (2020) found significant individual differences in cognitive functions in responses to acute physical activity by employing a mixed model. In addition, past experiences with sports and positive and negative feelings toward sports events possibly influence individual differences in response to physical activity. Taken together, individual variability in affective responses to acute physical activity may be even more pronounced than that observed in cognitive functions.

Given the previous research reviewed above, it is plausible that individual differences in valence and arousal in response to a single bout of physical activity exist. However, no study has quantitatively investigated the individual differences in core affect in response to physical activity. Therefore, this study aimed to quantitatively evaluate the individual differences in response to physical activity by decomposing the variance components. Quantifying the individual differences will help to elucidate what mechanisms and factors yield them, contributing to the practical tailoring of physical activity programs. For example, it may be possible to analyze the relationship between physical activity experience in childhood and individual differences in core affect in response to physical activity. Such results would bring practical information to researchers and experts in health promotion. The changes in valence and arousal before and after physical activity could have several variance components (Bourke et al., 2022; Kuppens et al., 2007; Timmermans et al., 2009). It was expected that individual differences are comprised of multiple variance components, including averaged variability as personality traits (Bourke et al., 2022; Timmermans et al., 2009), day-by-day variability (Kuppens et al., 2007; Timmermans et al., 2009), variability in response to acute physical activity, and random error. In this study, we employed a mixed model, which is also known as a multi-level modeling or a hierarchical linear model, to decompose the multiple variance components (Hedge et al., 2018; Nakagawa and Schielzeth, 2010).

This study hypothesized that individual differences in valence and arousal in response to acute physical activity are significant variance components. Furthermore, it was expected that the variance component of valence in response to acute physical activity would be more pronounced than that of arousal. The reason for expectation is that changes in valence before and after physical activity are influenced by multiple individual physiological and psychological traits, like anaerobic threshold (Ekkekakis et al., 2005), physical activity levels, subjective intensity (Farias-Junior et al., 2020), behavioral inhibition, and behavioral activation (Schneider and Graham, 2009). Additionally, emotional evaluation of likes and dislikes for specific sports events might also impact the valence during and after physical activity. On the other hand, the responses in arousal to physical activity would be linked to physiological variables such as increasing heart rate (HR) and blood pressure. Because variabilities of physiological variables during an identical physical activity are small and the reproducibility of those is adequate (Okin et al., 1986; Unnithan et al., 1995), individual differences in arousal in response to physical activity would be of a similar magnitude. Given the factors correlated with the response in valence and arousal, it was expected that valence exhibits greater interindividual variability than arousal. In addition, changes in the performance of processing speed in cognitive functions by the mixed model were also analyzed to compare variance components of processing speed with those of valence and arousal. Since no study has reported individual differences in response to physical activity as variance components, a reference value was needed to comprehensively evaluate the variance components of valence and arousal. Therefore, the individual differences in valence and arousal in response to physical activity were evaluated by comparing processing speed as the reference value.

## 2 Methods

### 2.1 Analyzed dataset

The dataset^1^ from our previously published study (Takahashi and Grove, 2023) was used. The previous study investigated the differences in acute effects of open-skill physical activity (badminton) and closed-skill physical activity (running) on executive function with a cross-over within-subjects research design. The study protocol was approved by the Human Subjects Committee of Tohoku Gakuin University (Approval number: 2019R003).

The previous study (Byun et al., 2014) measured valence and arousal as confounding variables for the association between executive function and physical activity. Although the original dataset includes valence and arousal before and after a control intervention (10 min of seated rest), the measurements of the control intervention were excluded from analyses in the present study to clarify the influence of physical activity on valence and arousal. The details of the experimental procedures of the dataset were described in the previous study. The experiment procedures related to this study are outlined below.

#### 2.1.1 Participants

The participants were healthy Japanese twenty-four undergraduate students (women was 9, age = 20.4 ± 0.2 years old, height = 168.2 ± 1.7 cm, weight = 61.6 ± 1.8 kg). A power analysis by G*Power version 3.1.9.4 software package (Düsseldorf, Germany), was conducted under the following conditions: the dependent variable was the change of measurements from pre-intervention to post-intervention; the independent variable was intervention with two levels: badminton, and running; partial eta squared was 0.1 (*f* = 0.33); power (1 − *β*) was 0.80; *ρ* was 0.5 and *α* was 0.05. This analysis indicated that the sample size of 20 was adequate, ensuring that the sample size *N* = 24 was valid. The participant’s criteria were (1) right-hand dominant undergraduate students, (2) normal or corrected to normal vision, and (3) no history of brain, cognition, mental, or cardiovascular diseases. The mean ± standard errors of the mean (SE) of the peak of oxygen consumption (VO_2_peak) and the peak of heart rate (HRpeak) for a graded running test on a motor-driven treadmill (O2road, Takei Sci. Instruments Co., Niigata, Japan) were 46.9 ± 1.1 mL·kg^−1^·min^−1^ and 192.9 bpm ± 1.8 bpm, respectively. The time spent on moderate-to-vigorous physical activity for a week was 2719.7 ± 652.2 min.

#### 2.1.2 Experimental procedures

Participants visited a sports physiology laboratory for 4 days (average interval, 6.1 ± 1.8 days). On the first visit, participants were given research guidance and signed the informed consent form. In order to familiarize the participants with a computer-based Stroop color-word test, which was a test to assess executive function, they conducted the Stroop color-word test twice. Subsequently, they underwent the graded exercise test. For the graded exercise test, the participants began running at 7.2 km·h^−1^ on the treadmill with the 1.0% slope. The running speed was increased by 1.2 km·h^−1^ every 2 min until they reached volitional exhaustion. A portable indirect calorimetry (MetaMax-3B; Cortex, Leipzig, Germany) recorded oxygen consumption (VO_2_), carbon dioxide output (VCO_2_), and heart rate (HR), and the average of the final 30 s was defined as VO_2_peak and HRpeak. The criteria of volitional exhaustion were (1) RPE ≥ 17, (2) HR ≥ 95% of age-predicted HRmax (220 minus age), and (3) a respiratory exchange ratio (RER, VCO2·VO2^−1^) ≥ 1.10.

On the second to fourth visit, the participants completed 10 min of badminton, running, or control interventions (one intervention per day). The sessions were conducted at consistent times of day within a ± 1-h window across participants. The order of each intervention was randomized, including the control condition. VO_2_, VCO_2_, and HR during each intervention were monitored by the indirect calorimetry. Before and 15 min after the interventions, the levels of valence and arousal were measured by the two-dimensional mood scale (Sakairi et al., 2013). After measuring the valence and arousal, they conducted the Stroop color-word task, which was composed of neutral and incongruent tasks. While incongruent tasks in the Stroop task are known as an index of executive function, neutral tasks that do not require executive function are recognized as an index of processing speed (Etnier and Chang, 2009). Each neutral and incongruent task included 24 trials, respectively, and each task’s average reaction time and accuracy rates were recorded. During the Stroop test, they were equipped with a functional near-infrared spectroscopy device to monitor hemodynamics in the prefrontal cortex.

The average reaction times for the neutral task were employed as the reference value but not measures for the incongruent task. Incongruent task performance was not employed because the previous study (Takahashi and Grove, 2020), which analyzed variance components in the incongruent task performance, could not detect a variance component representing individual differences in response to physical activity by multicollinearity.

#### 2.1.3 Interventions

In the badminton intervention, each participant played single games against a PE teacher on a standard badminton court (13.41 meters x 6.10 meters). All of the participants were novices in badminton. The PE teacher, who was not a professional in badminton, played with the participants and gave them some tips for performance improvements with social interactions. The scores were not recorded at the game, and participants and the PE teacher played the game recreationally. In the running intervention, participants ran at an estimated 75%VO_2_peak speed on the treadmill. The duration of both interventions was set to 10 min. During the treadmill intervention, participants were monitored by an experimenter to ensure safety, but no verbal communication occurred apart from safety checks. According to the previous study, the exercise intensities (%VO_2_peak and %HRpeak) were equivalent for running and badminton, and both interventions were vigorous physical activity (%VO_2_peak was about 77% and %HRpeak = about 85%) (ACSM, 2021).

#### 2.1.4 Assessment for valence and arousal

Levels of valence and arousal were measured using the two-dimensional mood scale (Sakairi et al., 2013). The two-dimensional mood scale was composed of eight items (6 Likert scales) that respond to the subjective feeling of “energetic,” “lively,” “lethargic,” “listless,” “relaxed,” “calm,” “irritated,” and “nervous.” The range of valence and arousal assessed by the two-dimensional scale was −20 to 20 points. For the valence, −20 points are “unpleasant,” and 20 points are “pleasant.” For the arousal, −20 points are “calmness,” and 20 points are “excitement.” Spearman-Brown’s coefficients of valence and arousal measured by the two-dimensional mood scale were more than 0.89, ensuring that the two-dimensional mood scale has adequate reliability. Sakairi et al. (2013) also demonstrated that the two-dimensional mood scale has sufficient factorial and discriminant validity by structural equation modeling.

### 2.2 Statistical analyses

A mixed model was employed using IBM SPSS 27 (SPSS Inc., Chicago, IL, United States) to estimate multiple variance components. The following equation (Equation 1) was fitted to the dataset.


(1) $$y_{\mathrm{ijk}}=\mu +\alpha_{j}+\beta_{k}+(\alpha \beta_{\mathrm{jk}})+b_{i}+{\left(\mathrm{b\alpha}\right)}_{\mathrm{ij}}+{\left(\mathrm{b\beta}\right)}_{\mathrm{ik}}+e_{\mathrm{ijk}}$$

where, *y_ijk_* is the points of valence, arousal, or processing speed of participant *i* = 1,…, *I* observed on the day of interventions *j* = 1,…, *J* at time point *k* = 1,…, *K*, with *μ* the grand mean, α*_j_* the fixed effect of the interventions, β*_k_* the fixed effect of time, (αβ)*_jk_* the fixed effect of the interaction of interventions and time, *b_i_* ∼ *N*(0, σ_p_^2^) the random effect of the participants, (*b*α)*ij* ∼ *N*(0, σ_pd_^2^) the random effect as the interactions between the participants and the day of interventions, (*b*β)*_ik_* ∼ *N*(0, σ_pt_^2^) the random effect as the interaction between the participants and time, and *e_ijk_* ∼ *N*(0, σ*_e_*^2^) the residual. The restricted maximum likelihood estimated the parameters in Equation 1.

To compare the variance component in response to physical activities for valence, arousal, and processing speed, the intraclass correlation coefficients (ICCs) were calculated according to the methods reported in previous studies (Brouwer et al., 2012; Demetrashvili et al., 2016).


(2) $$\mathrm{ICC}=\frac{\sigma_{\mathrm{pt}}^{2}}{\sigma_{\mathrm{pt}}^{2}+\sigma_{\mathrm{pd}}^{2}+\sigma_{e}^{2}}$$

In Equation 2, the numerator is the variance component of the interaction between the participants and time (σ_pt_^2^). It represents the individual differences in valence, arousal, or processing speed in response to physical activity. The denominator is the sum of the variance components without the variance component of the participants (σ_p_^2^). The variance of the interaction between the participants and the day (σ_pd_^2^) represents the individual differences in day-by-day variabilities for each measure. In calculating the ICCs, the variance σ_p_^2^ was not used because the variance represents the individual traits across the whole experimental procedure and is unrelated to the response to the physical activity. Based on the labels mentioned in Shrout (1998), ICCs were assessed as follows: “substantial” is 0.81–1.00; “moderate” is 0.61–0.80; “fair” is 0.40–0.60; “slight” is 0.10–0.40; “virtually none” is 0.0–0.10. The 95% confidence intervals (95%CIs) of the ICCs were estimated following the *F*-distribution approach by Demetrashvili et al. (2016).

## 3 Results

### 3.1 Fixed effects

**Figure 1:**
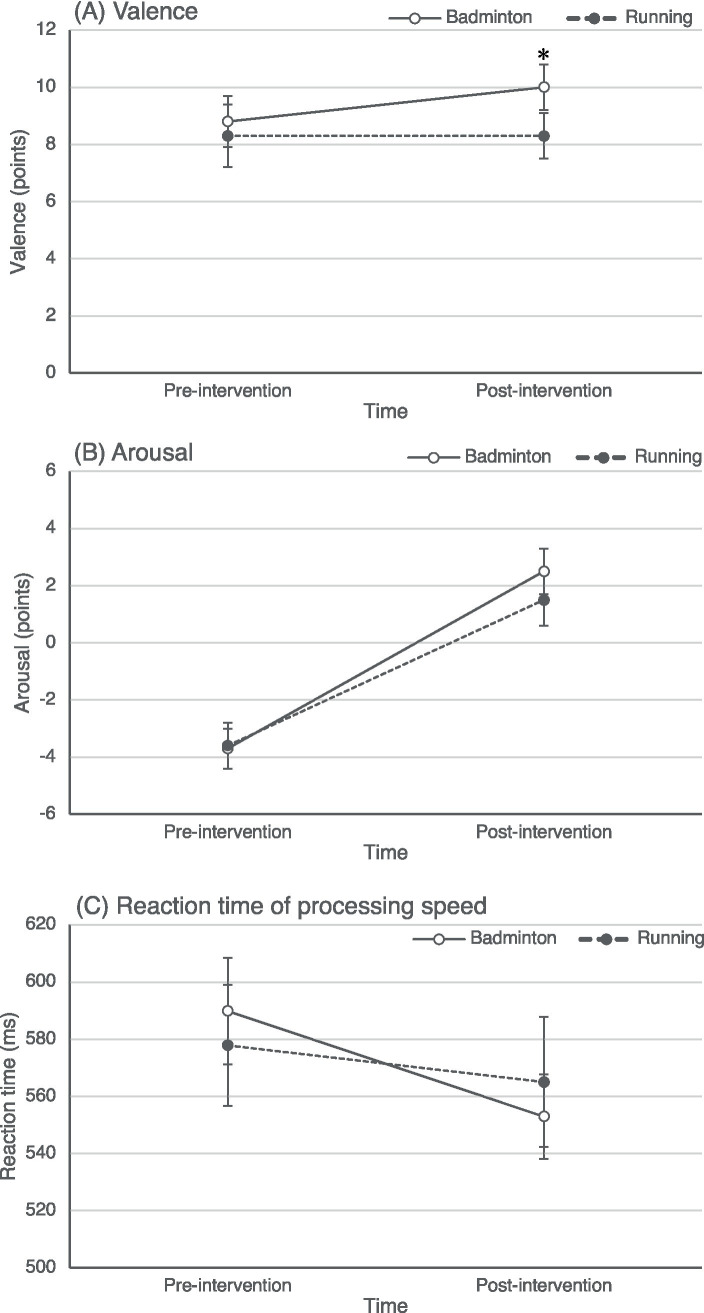
Changes in valence **(A)**, arousal **(B)**, and reaction time (RT) of processing speed **(C)** from pre- to post-intervention, respectively. Open circles with solid lines show the means of the badminton intervention, and closed circles with dashed lines show the means of the running intervention. Error bars show the standard error of the mean. Asterisk (*) represents a significant interaction, indicating that the increase of valence for the badminton intervention was significantly higher than that for the running intervention.

Figure 1 shows the valence, arousal, and processing speed changes for each intervention. The means ± SEs of the valence were 8.8 ± 0.9 points at pre-intervention and 10.0 ± 0.8 points at post-intervention for badminton, and 8.3 ± 1.1 points at pre-intervention and 8.3 ± 0.8 points at post-intervention for running, respectively. Regarding the arousal, the respective values were −3.7 ± 0.7 points at pre-intervention and 2.5 ± 0.8 points at post-intervention for badminton, and −3.6 ± 0.8 points at pre-intervention and 1.5 ± 0.9 points at post-intervention for running. The reaction times for information processing were 589.8 ± 18.7 ms at pre-intervention and 552.9 ± 14.8 ms at post-intervention for badminton, and 577.8 ± 21.2 ms at pre-intervention and 565.0 ± 22.8 ms at post-intervention for running, respectively.

Examining the changes in valence, the mixed model revealed a significant interaction of intervention and time (*F* (1, 23) = 4.7, *p* = 0.040). The badminton intervention significantly increased valence by 1.3 ± 0.6 points relative to the running intervention. For arousal, the main effect of time was significant (*F* (1, 34.6) = 42.3, *p* < 0.001); arousal at 15 min after interventions was significantly increased in 5.1 ± 1.0 points from pre-intervention. In contrast, the interaction of intervention and time (*F* (1, 23.6) = 0.9, *p* < 0.342) and the main effect of the intervention (*F* (1, 27.7) = 0.5, *p* = 0.485) did not significantly impact arousal. For processing speed, the mixed model revealed a significant main effect of time (*F* (1, 23) = 4.8, *p* = 0.040), but neither the interaction of intervention and time (*F* (1, 23) < 0.1, *p* = 0.995) nor the main effect of intervention (*F* (1, 23) = 2.6, *p* = 0.117) were significant. Both activity interventions significantly enhanced the processing speed.

### 3.2 Random effects and ICCs

Table 1 shows the estimated variances of random effects by the mixed models. For valence, the interaction between the participants and the day of interventions, and the interaction between the participant and time were significant, whereas the variance of the participant was not significant. For arousal, it was found that when repetitive calculations were performed to estimate variance components, the variance of the participant gradually transited to the variance of the interaction between the participant and time (Supplementary Table S1). Finally, the variance of participants was estimated at 0.0. This result might be caused by multicollinearity between σ_p_^2^ and σ_pt_^2^. A person with a lower arousal level at pre-intervention might tend to increase the arousal levels by physical activities in comparison to others with a higher arousal level at pre-intervention. The interaction between participants and the day of interventions was not significant, while the interaction between participants and time was significant. For processing speed, the variance of the participants and the interaction between the participants and the day of interventions were significant, but the variance of the interaction between the participants and time was not significant.

**Table 1:** Random effects from the mixed model.

Core affect	Random effects	Estimated variance ± SE
Valence	Participants (σ_p_^2^)	7.4 ± 4.1
	Participants × days of interventions (σ_pd_^2^)	2.8 ± 1.2*
	Participant × time (σ_pt_^2^)	7.3 ± 2.5*
	Residual (σ*_e_*^2^)	2.0 ± 0.6*
Arousal	Participants (σ_p_^2^)	NA
	Participants × days of interventions (σ_pd_^2^)	2.2 ± 1.9
	Participant × time (σ_pt_^2^)	5.2 ± 2.3*
	Residual (σ*_e_*^2^)	7.5 ± 2.2*
Information processing	Participants (σ_p_^2^)	3984.6 ± 1945.1*
	Participants × days of interventions (σ_pd_^2^)	3027.0 ± 1104.5*
	Participant × time (σ_pt_^2^)	896.9 ± 498.6
	Residual (σ*_e_*^2^)	1319.8 ± 389.2*

SE represents standard error of estimate, NA represents “Not available,” and * indicates that variance is significantly larger than 0.0 by the Wald test.

Table 2 shows the ICCs for valence, arousal, and processing speed in response to physical activity. The ICC of valence was evaluated as “fair.” The ICCs of arousal and information processing were evaluated as “slight.” Comparison between the ranges of 95%CIs showed that the ICC of valence was significantly more pronounced than the ICC of processing speed. On the other hand, the range of 95%CI for the ICC of arousal and the ICC of processing speed overlapped, so a significant difference between arousal and processing speed was not detected.

**Table 2:** ICCs for responses in valence and arousal to physical activity.

Core affect	ICC (95%CI)
Valence	0.603 (0.430–0.769)*
Arousal	0.349 (0.202–0.512)
Processing speed	0.171 (0.164–0.217)

ICC represents intraclass correlation coefficient, 95%CI represents 95% confidence interval, and * represents a significant difference from processing speed.

## 4 Discussion

### 4.1 Main findings

One of the goals of this study was to quantitatively evaluate individual differences in valence and arousal in response to physical activity. The mixed models found significant variance components for valence and arousal in response to physical activity. These results support this study’s hypothesis that the individual differences in core affect in response to acute physical activity are significant, elucidating that the acute effects of physical activity on feelings of affect and arousal vary among individuals. In addition, it was also hypothesized that individual differences in valence in response to physical activity are more pronounced than those in arousal. The ICC of valence was moderately more pronounced than the ICC of arousal. Additionally, the valence’s ICC was significantly greater than the processing speed’s ICC, whereas no significant difference in ICCs was found between arousal and processing speed. These results support this study’s hypothesis, consistent with previous studies’ findings (Ekkekakis et al., 2005; Farias-Junior et al., 2020; Schneider and Graham, 2009) that reported valence changes influence multiple physiological and psychological traits. The results also support the speculations by Reed and Ones (2006). Because high valence and arousal states, such as enjoyment and enthusiasm, are critical determinants of long-term physical activity adherence (Huberty et al., 2008; Rhodes et al., 1999), the findings of this study underscore the importance of considering individual differences in valence and arousal when tailoring physical activity programs.

### 4.2 Validity of dataset

The mixed model found a significant interaction between the interventions and time for the valence measures, indicating that badminton increased valence levels more than running. For arousal, while the main effect of time was significant, the interaction between the interventions and time and the main effect of the interventions were not significant, showing that the badminton and running interventions equally increased arousal. Playing badminton likely enhances positive and activated affect compared to running. Given that exercise intensity and duration (10 min) were equivalent for both interventions, badminton’s positive effect might come from social factors. Each participant played with the experimenter (PE teacher) for the badminton intervention, while the participants ran alone on the treadmill for the running intervention. The badminton intervention included social interactions, but the running intervention did not. Exercising alone or with others with social interaction potentially brings different effects on executive function and mental health (Seino et al., 2019; Nagata et al., 2023; Pesce et al., 2009). The results, in which badminton enhanced valence and arousal more than running, are consistent with the perspective from the previous studies, ensuring that the dataset was valid to investigate the association between physical activity and feelings. Although the results support the validity of the dataset, this experimental design may not be ideal for investigating the effects of social interaction on affect. Due to other potential confounding factors (e.g., various movements such as swinging or jumping, and visuomotor demands), further studies with more controlled designs are needed to examine the influence of social interaction.

On the other hand, the different effects of badminton and running should be interpreted carefully. As described above, the badminton intervention showed a significantly greater effect on affect than the running intervention. However, the previous study (Takahashi and Grove, 2023) did not find differences in the changes in valence and arousal before and after the interventions by including the control intervention (10 min seated rest with manipulating smartphone) with a mixed model. Given that adding measurements from the control intervention failed to find the significant differences in the physical activity types on core affect, the difference between badminton and running does not appear crucial for daily life.

### 4.3 Individual differences in response to physical activity

This study is the first to quantitatively investigate individual differences in valence and arousal in response to physical activity. The multiple variance components, individual traits in participants across the whole experimental procedure (σ_p_^2^), individual differences in day-by-day variability (σ_pd_^2^), and individual differences in response to physical activity (σ_pt_^2^) were decomposed using a mixed model. For valence, σ_pt_^2^ was a significant variance component and independent of σ_p_^2^. Given that the variance of σ_p_^2^ represents how much each participant’s valence varies in the experimental procedures, the σ_p_^2^ is the same as the within-person variabilities in previous studies (Kuppens et al., 2007; Timmermans et al., 2009). The previous studies reported that the within-person variability of core affect correlates with personality traits and impacts mental health. Taken together with the findings of this study and those of previous studies, this study’s results suggest that individual differences in valence in response to physical activity is not only caused by personality traits but also by other factors. Given previous studies’ findings, which indicate that anaerobic threshold, usual physical activity levels, behavioral inhibition, and behavioral activation impact changes in valence to physical activity (Ekkekakis et al., 2005; Farias-Junior et al., 2020; Schneider and Graham, 2009), past experience of success or failure in sports performance, individuals’ usual physical training status and its results, aerobic and anaerobic metabolic features might be responsible for individual differences. Genetic factors could also be the cause. Neurotrophic factors such as brain-derived neurotrophic factor and insulin-like growth factor, which are raised by acute physical activity (Behrendt et al., 2021; Hung et al., 2018), are known to benefit psychological status (Deyama et al., 2022). Therefore, genetic factors linked to neurotrophic factors could also influence individual differences in valence in response to physical activity. Elucidating what factors determine individual differences in valence in response to physical activity would lead to a more efficient tailoring of a physical activity program to enhance health. Future research should investigate the causes of individual differences in valence in response to physical activity.

In contrast, the variance components in arousal showed different results for valence. Although the mixed model revealed a significant variance of σ_pt_^2^ in arousal, the variance of σ_p_^2^ was not detected. Repetitive calculations to estimate random effects in arousal imply that there is a possibility of multi-collinearity between the variances of σ_pt_^2^ and σ_p_^2^, suggesting that individuals with low arousal at the pre-intervention likely have more activated arousal after physical activities than those with high arousal at the pre-intervention. Unlike valence, the individual differences in response to physical activity on arousal could stem from not only feelings of likes and dislikes and sports or educational careers but also personality traits or genetic factors (Kuppens et al., 2007; Timmermans et al., 2009).

On the other hand, σ_pt_^2^ of processing speed was not significant. The results were consistent with the previous study (Takahashi and Grove, 2020). In the behavioral performance of cognitive tasks such as the Stroop tasks, the individual differences are much more extensive, but the changes in cognitive performance after a single bout of physical activity do not appear to vary among individuals. It is evident that the effects of acute physical activity on valence and arousal vary among individuals compared to cognitive functions. Understanding extensive individual differences in the impact of physical activity on affect seems important when considering the effects of physical activity on psychological variables.

### 4.4 ICCs of valence and arousal

Another goal of this study was to compare individual differences in response to physical activity on valence and on arousal. It was expected that the variance of σ_pt_^2^ in the valence would be more pronounced than in the arousal. ICCs that can relatively evaluate variances were calculated to compare valence and arousal. The ICCs of valence and arousal were evaluated as “fair” and “slight,” respectively. Furthermore, the ICC of valence was significantly greater than the ICC for processing speed, whereas a difference between the ICCs of arousal and information processing was not significant. These results agree with the hypothesis.

It was expected that with controlled intensities (%VO_2_peak and %HRpeak) and duration of physical activities across individuals, changes in physiological variables would not generally vary across individuals, leading to a relatively small variance of σ_pt_^2^ in arousal than in valence because the levels of arousal would be associated with physiological variables. As per the expectations, major changes in arousal by physical activity may be explained by exercise-induced physiological responses such as activation of the sympathetic nervous system and inhibition of the parasympathetic nervous system.

## 5 Limitations

This study had some limitations. First, the method of sampling the participants might bias the results. The dataset reported by Takahashi and Grove (2023) was analyzed. According to their study, they recruited participation through sports and health sciences courses in the university, and then 24 healthy undergraduate students voluntarily participated in the experiments. Considering the recruitment method, most participants likely have positive attitudes toward sports and physical activity. The attitudes might influence the results. A different pattern might be observed with a sample containing participants with negative attitudes toward sports and physical activity. For example, it might be expected that with more individuals with negative feelings toward sports and physical activity, the individual differences in core affect, especially valence, to a single-bout physical activity would be more pronounced than reported here. Second, this study analyzed only two types of physical activity. Both the badminton and running interventions were vigorous aerobic activities, and the durations were very short (10 min). Ekkekakis et al. reported that core affect in response to physical activity largely varies when exercise intensity is around the anaerobic threshold. Because the intensities of the badminton and running interventions (75%VO_2_peak) seem to be higher than anaerobic threshold levels, the variability of core affect in the present study could be reduced. When the exercise intensity approximates the anaerobic threshold level (moderate intensity), the individual differences in valence and arousal could be increased. Different findings could be obtained when physical activities with low or moderate intensities, anaerobic exercises such as resistance training, and more prolonged durations.

## 6 Conclusion

This study quantitatively evaluated individual differences in valence and arousal in response to acute physical activities. In conclusion, changes in valence and arousal to physical activity significantly vary among individuals. In particular, individual differences in valence were more pronounced than in arousal. The findings of this study suggest the importance of considering individual differences in feelings in response to physical activity when tailoring physical activity treatment for health. Tailoring interventions based on emotional responsiveness may lead to more effective, personalized health strategies. Future studies need to investigate mechanisms underlying these individual differences and explore how tailoring exercise interventions considered individuality in core affect would enhance psychological outcomes across broader populations.

## Data Availability

The original contributions presented in the study are included in the article/Supplementary material, further inquiries can be directed to the corresponding author.

## Footnotes

^1^ https://doi.org/10.1371/journal.pone.0276148.s001
