# Supplementary material for: Individual differences in affect in response to physical activity
Source: Front Psychol. 2025 Jun 10;16:1575189. doi: 10.3389/fpsyg.2025.1575189 (PMC12186705; doi:10.3389/fpsyg.2025.1575189)
Supplement: Supplementary file 1 [file Table_1.docx]

Supplementary Material

# Table S1. Repetitive calculations to estimate random effects in arousal.

| Iteration | -2 restricted likelihood | Participants (σ_p_^2^) | Participants × days of interventions (σ_pd_^2^) | Participants × time (σ_pt_^2^) | Residual (σ*_e_*^2^) |
| --- | --- | --- | --- | --- | --- |
| 0 | 527.649 | 3.731 | 3.731 | 3.731 | 3.731 |
| 1 | 517.496 | 1.614 | 3.083 | 4.666 | 5.560 |
| 2 | 515.882 | 0.026 | 2.930 | 5.008 | 6.112 |
| 3 | 515.271 | 0.000 | 2.437 | 5.252 | 7.071 |
| 4 | 515.226 | 0.000 | 2.260 | 5.240 | 7.455 |
| 5 | 515.226 | 0.000 | 2.240 | 5.230 | 7.499 |
| 6 | 515.226 | 0.000 | 2.240 | 5.230 | 7.500 |
| 7 | 515.226 | 0.000 | 2.240 | 5.230 | 7.500 |

**
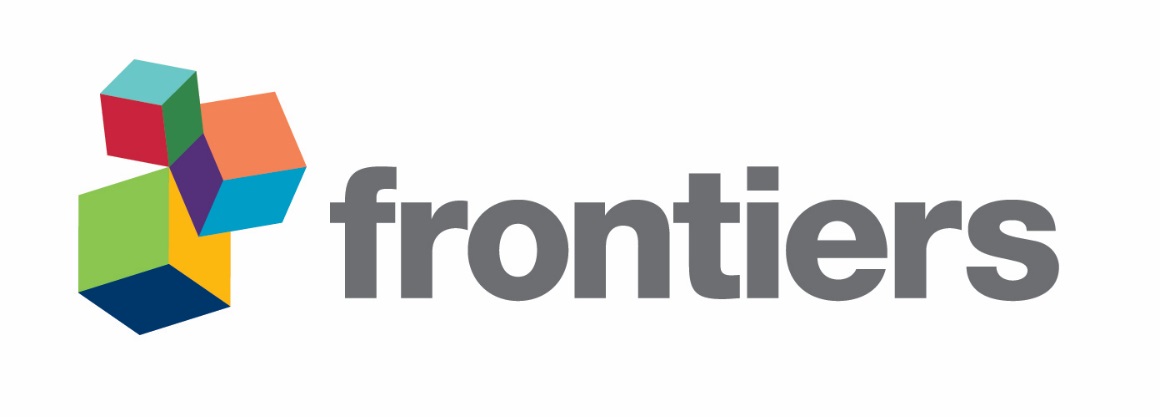
**
